# Biomechanics of the natural, arthritic, and replaced human ankle joint

**DOI:** 10.1186/1757-1146-7-8

**Published:** 2014-02-06

**Authors:** Alberto Leardini, John J O’Connor, Sandro Giannini

**Affiliations:** 1 Movement Analysis Laboratory, Istituto Ortopedico Rizzoli, Bologna, Italy; 2 University of Oxford, Oxford, England; 3 Department of Orthopaedic Surgery, Istituto Ortopedico Rizzoli, Bologna, Italy

**Keywords:** Tibiotalar, Subtalar, Ankle complex, Rear-foot, Articular surfaces, Ligaments, Osteoarthritis, Joint mobility, Joint stability, Gait analysis, Joint replacement, Arthrodesis

## Abstract

The human ankle joint complex plays a fundamental role in gait and other activities of daily living. At the same time, it is a very complicated anatomical system but the large literature of experimental and modelling studies has not fully described the coupled joint motion, position and orientation of the joint axis of rotation, stress and strain in the ligaments and their role in guiding and stabilizing joint motion, conformity and congruence of the articular surfaces, patterns of contact at the articular surfaces, patterns of rolling and sliding at the joint surfaces, and muscle lever arm lengths.

The present review article addresses these issues as described in the literature, reporting the most recent relevant findings.

## Background

**Figure 1:**
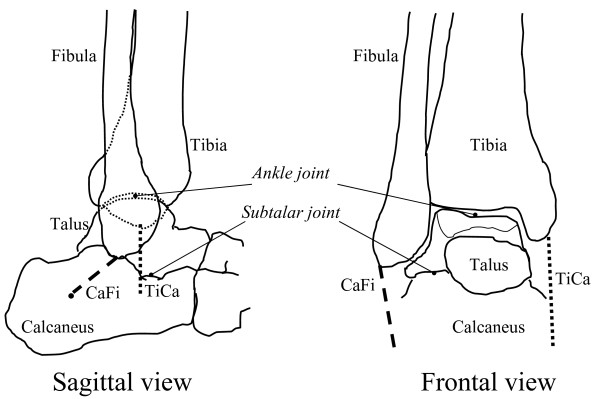
**Diagrams of natural anatomy.** Diagrammatic representation of the main bones, joints and anatomical structures. The location of the calcaneofibular (CaFi) and the tibiocalcaneal (TiCa) ligaments, important for following descriptions, is depicted.

The human shank and foot complex is an intricate, multi-joint mechanism, which is fundamental for the interaction between the lower limb and ground during locomotion. The ankle complex (Figure 1) mainly formed by the ankle (or tibiotalar) and subtalar (or talocalcanear) joints plays a fundamental role in the human locomotor system, being involved in virtually every locomotion activity. The inferior tibiofibular and fibulotalar joints also play a role in the ankle joint complex but this is not explicitly addressed in the present paper.

Motion at the ankle and subtalar joints is guided by the osteoarticular and ligamentous structures and induced by the forces and moments of the extrinsic muscles, in addition to the external forces. Muscles act by applying force to the bones through muscle tendons with instantaneous lever arms relative to the joint centre; at the ankle complex the tendons wrap around bones and change direction under retinaculae. The talus does not have tendon attachments, and is constrained by ligament and contact forces. Lever arm lengths determine the ability of muscles to produce joint torque in order to generate or resist rotation. Any injury, lesion or neuromuscular disorder of this complex system affects these interactions between muscles, bones and ligaments and causes degradation, instability or disability of locomotion. To enhance understanding of disorders and of relevant conservative and surgical treatments, a better knowledge of the physiological mechanics of the ankle complex still remains a crucial issue.

## Mobility and stability at the human normal and arthritic ankle joint

Joint replacement is necessary in severely arthritic ankles to reduce pain, to restore joint stability, and to restore joint mobility. Paradoxically, the first two goals can be achieved by a joint arthrodesis, therefore joint mobility is the primary goal of joint replacement. It is also a primary aim of ligament reconstruction. A disappointing range of movement in the replaced ankle joint often results from the continued presence of contracted soft tissue around the joint [1]. Rational design and surgical implantation of prostheses therefore demands understanding of the natural interactions between ligaments and articular surfaces of the two joints which control ankle complex mobility.

Stability, joint resistance to relative movement of the bones when load is applied, is also a key requirement of joint replacement. *Passive* stability, as assessed in a range of clinical tests, is a measure of the limitations to motion imposed by the anatomical structures and therefore involves mechanical interactions between ligaments and articular surfaces and reflects both the integrity of those structures and their mechanical properties [2]. *Active* stability involves mechanical interactions between muscles, ligaments and articular surfaces in response to external forces during activity.

Restoration of normal joint function and range of motion should benefit from re-establishment of the natural relationships between the geometry of the articular surfaces and the geometry of the ligaments [3]. The current separate practises of ligament reconstruction and joint replacement for the ankle suggests that these geometric relationships are not yet fully understood. Such understanding could lead to concurrent ligament reconstruction and joint replacement, when necessary.

Geometrical studies of human joints are aimed at showing how the ligament orientations and the shapes of the articular surfaces are able to guide the movements of the bones upon each other within their allowable range of movement. On the other hand, mechanical studies show how the ligaments can act together with the muscles and the articular surfaces to transmit load from one bone to the other within their allowable range of movement and how they combine to define that range of movement. Understanding of the role of all the passive structures in the natural ankle joint is important for a successful design of joint replacements which can fully restore original joint function. In particular, knowledge of the changing geometry of the passive structures throughout the range of passive flexion is necessary for a successful mechanical analysis of the response of the joint to external load. Joint mobility and stability depend also by these mechanisms.

In the following part of Section 1 of this review, we describe the natural mobility and stability of the human ankle complex and the inter-relationships between articular surfaces and ligament fibres. Erosions of the former caused by the various forms of arthritis, and degeneration of the latter caused by injury or joint deformation, generate instability of the ankle and subtalar joints and disability of the entire locomotion system in addition to pain. In the most severe of such cases, arthrodesis is still the current surgical treatment of choice [4,5], but arthroplasty has been investigated systematically as well since early 1970’s [6].

### Joint mobility in the normal ankle

Motion at the ankle joint complex has been divided into that at the ankle and at the subtalar joints [7,8]. Computer-tomography based stress-tests in-vivo in non-weight bearing conditions revealed that from maximal dorsi- to maximal plantar-flexion, the mean overall rotation over 20 healthy subjects is much higher at the ankle (63°) than at subtalar (4°) joint [9]. Much smaller difference was observed in the complete natural range from maximal combined eversion–dorsiflexion to maximal combined inversion–plantarflexion (49° at the ankle, 30° at the subtalar). During the stance phase of walking, the joint rotations in the three anatomical planes were found to be on average about 15°, 8°, and 8° at the ankle joint, and about 7°, 10°, and 7° at the subtalar joint [10].

Initially, combined motion at these two articulations was considered to be a rotation about a single or a double fixed axis [11-14]. Patterns of joint motion were investigated thoroughly but basically with the same assumption [15,16]. More recent studies have reported that the instantaneous axis of rotation translates and rotates during passive dorsi-plantar flexion [17,18], suggesting that the hinge joint concept is an oversimplification. A few recent works have also demonstrated an associated shift of the contact area during flexion not only at the trochlea tali but also at the tibial mortise [19]. In these joints therefore, rolling (revolving about the contacts) as well as sliding (gliding over the contacts) occurs, consistent with multiaxial rotation. An approximately isometric pattern of elongation throughout joint rotation was demonstrated [17,20] for two ligaments (Figure 1), the calcaneofibular (CaFi) and the tibiocalcaneal (TiCa: this ligament is used to describe the central superior fibres of the deltoid ligament on the medial side of the rear-foot ligaments); in other words, 3D rotation of the ankle joint takes place with minimal change in length in these two ligaments. Other ligament fibres were slack over most of the range of passive dorsi-plantar flexion and tightened only at one or other of the limits of motion. These findings suggest a close interaction between the geometry of the ligaments and the shapes of the articular surfaces in guiding and stabilising ankle joint motion. The following will develop this concept.

#### Experimental observations in-vitro

Experimental in-vitro work was performed by the present authors explicitly to investigate whether or not a preferred path of joint motion is prescribed by the passive joint structures alone during dorsi- plantar-flexion in virtually unloaded conditions [17]. This is fundamental knowledge for any design of relevant surgical treatments. A rig was built to move the ankle complex through its full range of flexion while applying a minimum load. Joint motion was constrained therefore only by the articular surfaces and the ligaments.

The movements of the calcaneus, talus and fibula relative to the stationary tibia in lower-leg preparations were tracked with a stereophotogrammetric system. It was shown that for each individual specimen, the calcaneus follows a unique path of unresisted coupled motion relative to the tibia and that most of this motion occurred at the ankle, with little motion at the subtalar level. The CaFi and the TiCa ligaments showed near-isometric pattern of rotations about their origins and insertions, whereas posterior ligaments slackened during plantarflexion and anterior ligaments slackened during dorsiflexion. In other words, during virtually unloaded joint movement, there are ligament fibres that maintain constant length throughout movement, and this must guide joint mobility, and others that tighten to define only the extremes of this movement.

All specimens showed motion of the axis of rotation relative to the bones. Perturbations from this unique path of passive motion induced by the application of load involved mostly subtalar joint motion and were resisted. The perturbations were completely recovered when the loads were removed, and the joint returned to its unique path of passive motion. Therefore, the ankle complex exhibits one degree of unresisted freedom. The subtalar joint complex behaves as a flexible structure which moves only because of soft tissue deformation when loaded [18]. Further experiments with higher resolution techniques, i.e. combination of roentgen-stereophotogrammetry and 3D digitisation, showed that the most anterior fibres within the CaFi and TiCa rotate most isometrically [20], i.e. experience the smallest strain, and that an anterior translation of the articular contact on the tibial mortise occurs during dorsiflexion [19]. It was deduced that the ankle is a single degree-of-freedom mechanism where passive mobility is allowed by rolling as well as sliding of the articular surfaces upon each other and by the isometric rotation of the two ligament fibres about their origins and insertions, therefore without major deformation of these tissue. In other words, in the absence of tissue deformation, passive motion is unresisted.

#### Corresponding mathematical models in the sagittal plane

**Figure 2:**
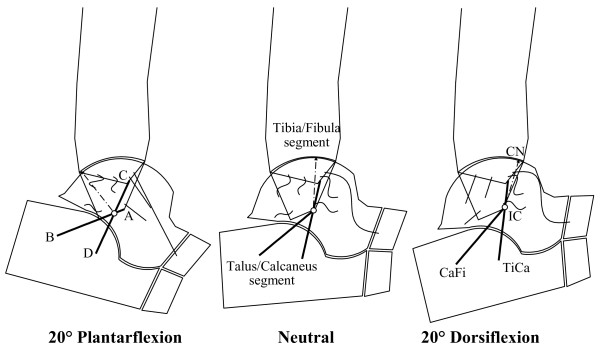
**4-bar-linkage model, single fibre ligaments.** Diagrammatic sketches of the single-degree-of-freedom mechanism in the sagittal plane as predicted by the geometrical the 4-bar-linkage model. The geometrical arrangement of the passive structures is shown in three joint positions: at 20° plantarflexion (left), neutral (central) and 10° dorsiflexion (right). The kinematics is guided by the isometric rotation of the CaFi and TiCa ligaments (solid bold). The articular surfaces (the arcs nearly in contact), the line contact, i.e. the common normal CN at the single contact point, the other ankle ligaments (buckled segments), and the instantaneous centre of rotation IC (empty circle) are also depicted.

**Figure 3:**
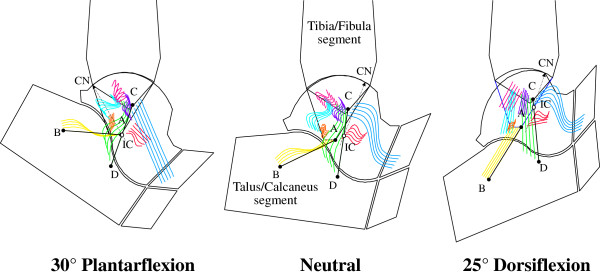
**4-bar-linkage model, with fibre recruitment.** Diagram similar of Figure 2, but with the model representation of ligament fibres as array of line segments; the pattern of fibre recruitment over flexion is depicted by the buckling of the ligament fibres.

Computer-based geometrical models [21] elucidated this mechanism, initially in the sagittal plane (Figure 2), where most of the passive motion was shown to occur. Fibres within the CaFi and TiCa ligaments rotate isometrically about their origins and insertions (a four-bar-linkage when projected in the sagittal plane), while all other ligament fibres located more anteriorly slacken during dorsiflexion, those located posteriorly slacken during plantarflexion (Figures 2 and 3), becoming tight just at the corresponding extremes.

The instantaneous centre of rotation (IC), the point at which the two isometric ligament fibres cross in the plane, moves from a postero-distal to an antero-proximal position during dorsiflexion. The articular contact point, depicted in Figure 2 by the common normal (CN), moves from the posterior part of the tibial mortise in maximal plantarflexion to the anterior part in maximal dorsiflexion so that the talus rolls forwards while sliding backwards on the tibial mortise during dorsiflexion, and it rolls backwards while sliding forwards on the mortise during plantarflexion.

The shapes of the articular surfaces must be compatible with this ligament rotation, i.e. articular surfaces must move in contact with one another while maintaining these fibres just tight at constant length. The deduced shape of the complementary surface of the talus, compatible with a mortise shape taken as an arc of a circle, is a polycentric and polyradial curve as in the intact talus.

The model was then extended by including arrays of fibres for each ligament (Figure 3). The mechanical effect of the extensor retinaculae was included to predict the changing lever arm lengths of the main flexor and extensor muscles [22], calculated as the perpendicular distances from the IC to each tendon. Figure 3 shows that the changing positions of both muscle lines of action and of the instantaneous centre of rotation produce a lever arm of the flexor muscles maximised in dorsiflexion, and that of the extensor muscle maximised in plantarflexion. The joint positions in which these two muscle groups fire during gait are exactly those in which they were predicted to be mechanically advantaged.

#### Equivalent spatial mechanisms

**Figure 4:**
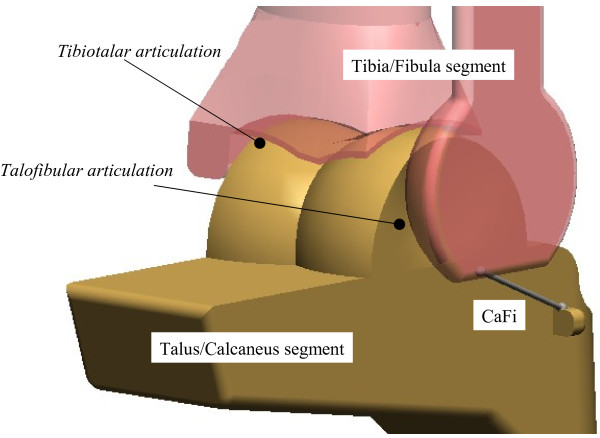
**Diagram of a 3D model.** Diagram of a three-dimensional geometrical model for ankle joint mobility.

Three-dimensional (3D) computer-based geometrical kinematic models of the tibiotalar articulation were subsequently developed to explain the multi-axial coupled motion observed experimentally during passive motion [23,24]. Two one-degree-of-freedom spatial equivalent mechanisms for the tibiotalar joint passive motion simulation were initially proposed [23]. The mechanisms were based on the assumption of the guiding role of the joint passive structures, such as ligaments and articular surfaces, and on their geometric dimensions. These assumed isometricity of fibres within the calcaneofibular and tibiocalcaneal ligaments and rigidity of the articulating surfaces, taken as three sphere-plane contacts in one model (Figure 4), and as a single spherical pair in the other one.

Although motion predicted by the models was reasonably compatible with that measured in corresponding specimens, considerable differences were observed. This was accounted for by the oversimplifications adopted to represent the anatomical structures, particularly the complex articular surfaces in spheres and planes. Later, a surgical navigation system with cluster of active markers was used to collect more precise skeletal kinematics and anatomical geometry of the passive structures, i.e. articular surfaces and attachment areas of the ligaments, by digitisation with a pointer [24]. An equivalent spatial mechanism for the passive motion simulation was defined by three sphere-to-sphere contact points and two rigid links. These contact points were identified at the lateral talofibular articulation and two at the articulation between tibial mortise and trochlea tali. The two rigid links were identified by the isometric fibres at the calcaneofibular and tibiocalcaneal ligaments. An optimisation algorithm was developed for the identification of the final geometrical parameters resulting from an iterative refining process, which targets best matching between model predictions and corresponding experimental measurements of the spatial motion.

The specimen-specific equivalent spatial mechanisms replicated the original passive motion from corresponding specimens very well. The study demonstrates further that the articular surfaces and the ligaments, acting together as a mechanism, control the passive kinematics of the ankle joint in a complex 3D path of motion. In particular, it was demonstrated that the passive structures of the ankle joint alone are able to guide the complex triplanar motion, where the about 45° flexion in the sagittal plane is coupled with about 4-5° and 7-8° rotations respectively in the frontal and transverse planes.

During activities of daily living, the ankle functions under load. In response, ligaments stretch or slacken and articular surfaces in contact indent. The passive motion models here above define the initial configuration of these joint structures at each flexion angle, from which the final configuration under load can be calculated incrementally, as described in the following paragraphs.

### Mechanical models of ankle joint stability

An objective of musculo-skeletal biomechanical studies is a thorough understanding of joint stability as well as joint mobility. Little has been reported in the literature about stability, probably because of limited knowledge of mobility, in particular the synergic role of the ligaments and articular surfaces. It has been recognised that only a limited percentage of ankle joint translational and rotational stability can be accounted for by geometry of articular surfaces [25]. Although we are far from a comprehension of the complexities of ankle and rearfoot joint stability during activities of daily living, preliminary valuable findings were reported for the elementary, though clinically relevant, drawer test.

#### Anterior drawer test

Experimental and modelling work on fibre recruitment, in both 2D and 3D, provides information to interpret elementary mechanical tests routinely used for clinical assessments, in particular the anterior drawer test. At each joint position within the flexion arc, the ligament structures which resist the external force change not only orientation, but also the thickness, because of the progressive recruitment and tightening of fibres (Figure 3; see also [20]. This is one of the possible explanations of the observation that the resistance to load of the natural ankle varies along its flexion arc [26-28].

Mathematical models of the ankle joint were developed to study ligament fibre recruitment and to calculate relevant load/displacement curves at different flexion angles within the passive flexion arc [29,30]. Ligaments were modelled as 3D arrays of fibres, though their orientations at different flexion angles were taken from the four-bar-linkage model in the sagittal plane [21]. A non-linear stress/strain relationship was assumed for ligament fibres and relevant mechanical parameters were taken from the literature. Talus and calcaneus were assumed to move as a single rigid body. The antero/distal translational motion of the talus relative to the tibia was calculated.

The ankle joint was found to be stiffer at the two extremes of the flexion range, and the highest laxity was found around the neutral position, confirming previous experimental works. In a first paper [29] the quantitative comparison between model predictions and experimental measurements was not fully encouraging, because of the elementary nature of the datasets used for the mechanical parameters of the ligaments. In a second paper [30], the anterior drawer test was assessed also considering the effect of ligament viscoelasticity on the force response of the ankle joint, and a third data set [31]. The stiffness of the model ankle joint increased only modestly with velocity. The response force found for a 6 mm displacement at plantarflexion increased by only 13% for a one hundred-fold increase in velocity from 0.1 to 10 mm/s. The model predictions agreed well with the same experimental results cited above. The flexion angle was confirmed as the most influential parameter in the mechanical response of the ankle to anterior drawer test, supporting further the view that the comprehension of joint mobility is a necessary prerequisite for the comprehension of joint stability.

### Function of the foot in gait

**Figure 5:**
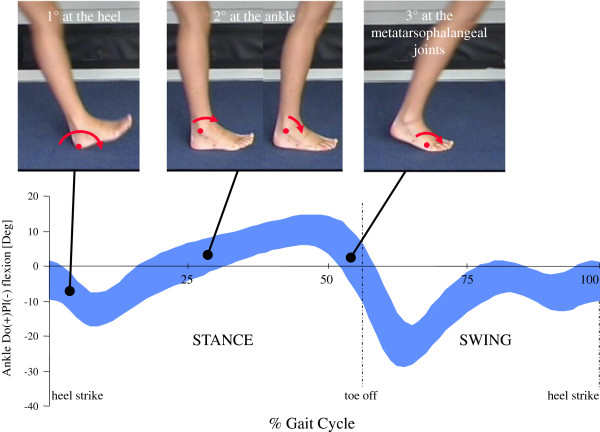
**Mechanism of the three rockers of the foot.** Mechanism of the three rockers of the foot-to-shank motion; about the heel first, the ankle second, and the first metatarsophalangeal joint third; the corresponding pattern of dorsi- plantar-flexion is plotted over the gait cycle in level walking is also shown.

**Figure 6:**
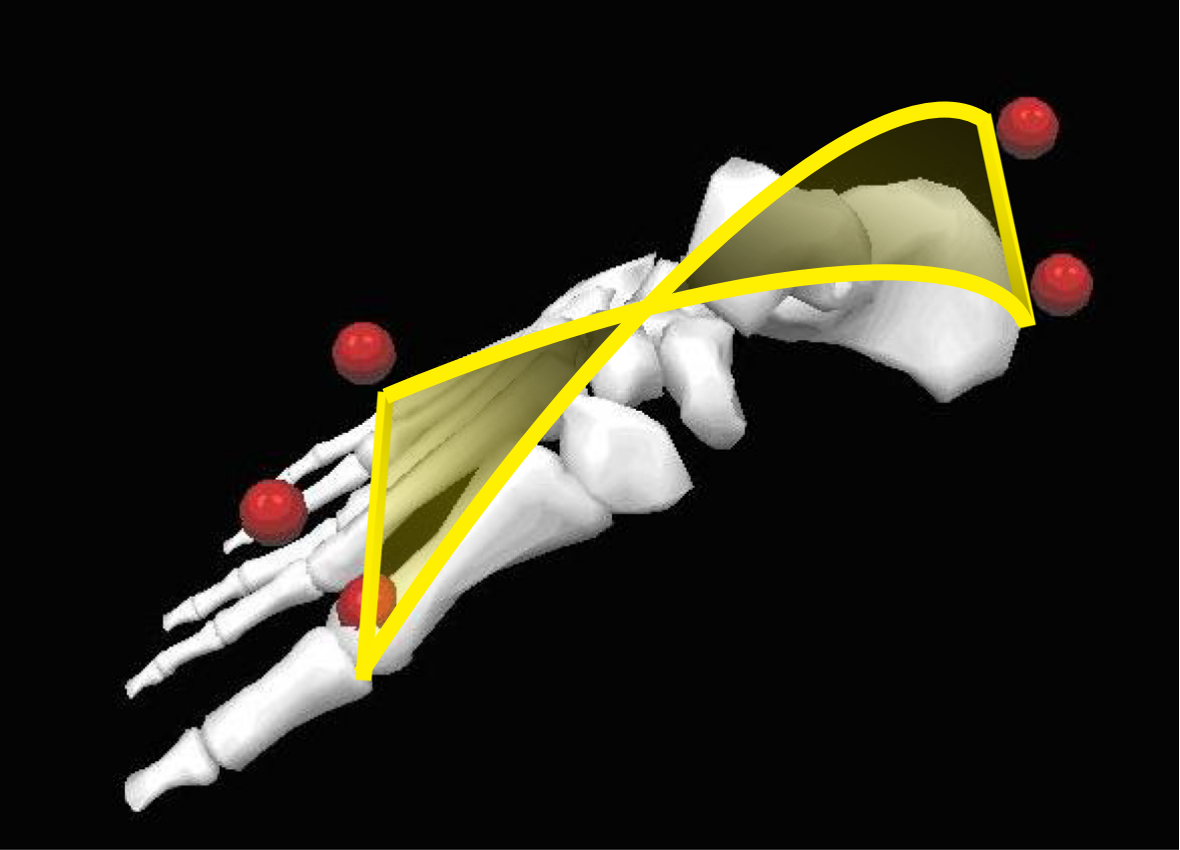
**‘Elica podalica’.** A graphical representation for the concept of ‘elica podalica’, originally rearrangement after Paparella Treccia 1978 [35].

The ankle and subtalar joints analysed so far are only the connecting part of the even more complex foot segment, which is fundamental in human locomotion. The foot and ankle unit provides the three rockers of the walking cycle [32], i.e. three different rotations in the sagittal plane about three different points (Figure 5): 1) about the heel in contact with floor, from the terminal part of the swing phase until the foot is flat on the ground, it controls the lowering of the foot to the floor; 2) about the ankle joint, during the period in which the foot remains flat on the ground and the shank advances, it controls the continued forward movement of the body; and 3) about the metatarso-phalangeal joints, during the push-off phase, it allows the generation of power for progression of the relevant limb [33]. During each of these phases, either the entire foot becomes flexible in response to loading or stiffens to favour propulsion [34]. For these phenomena, considerable and complex motion occurs at the many foot joints; in the literature, these mechanisms have been analysed and presented as ‘shock absorption’, ‘navicular drop’, ‘windlass mechanism’, ‘foot clearance’, ‘elica podalica’ (helical airscrew between the rear- and fore-foot, Figure 6) etc. These complex mechanisms at the foot have been investigated in-vivo by using many different techniques, as briefly discussed in the next Section.

## Biomechanics of gait at the human ankle complex

Because of its fundamental role and complex function, thorough assessment of foot pathology during walking should form an integral part of every clinical evaluation [36]. The mere observation of gait cannot detect and quantify subtle motion of the single bones and deformation of the entire foot segment, therefore quantitative 3D gait analysis is necessary to provide information on the dynamic function of the foot and to contribute to the assessment of relevant treatments; total ankle replacement for example is addressed in the present paper. Reliable assessment of gait and other activities of daily living, performed before and after surgery or pharmacology, is necessary to establish quantitatively the efficacy of treatments aimed at improving function at the foot and ankle complex.

### Methods for tracking foot and ankle motion in-vivo

**Figure 7:**
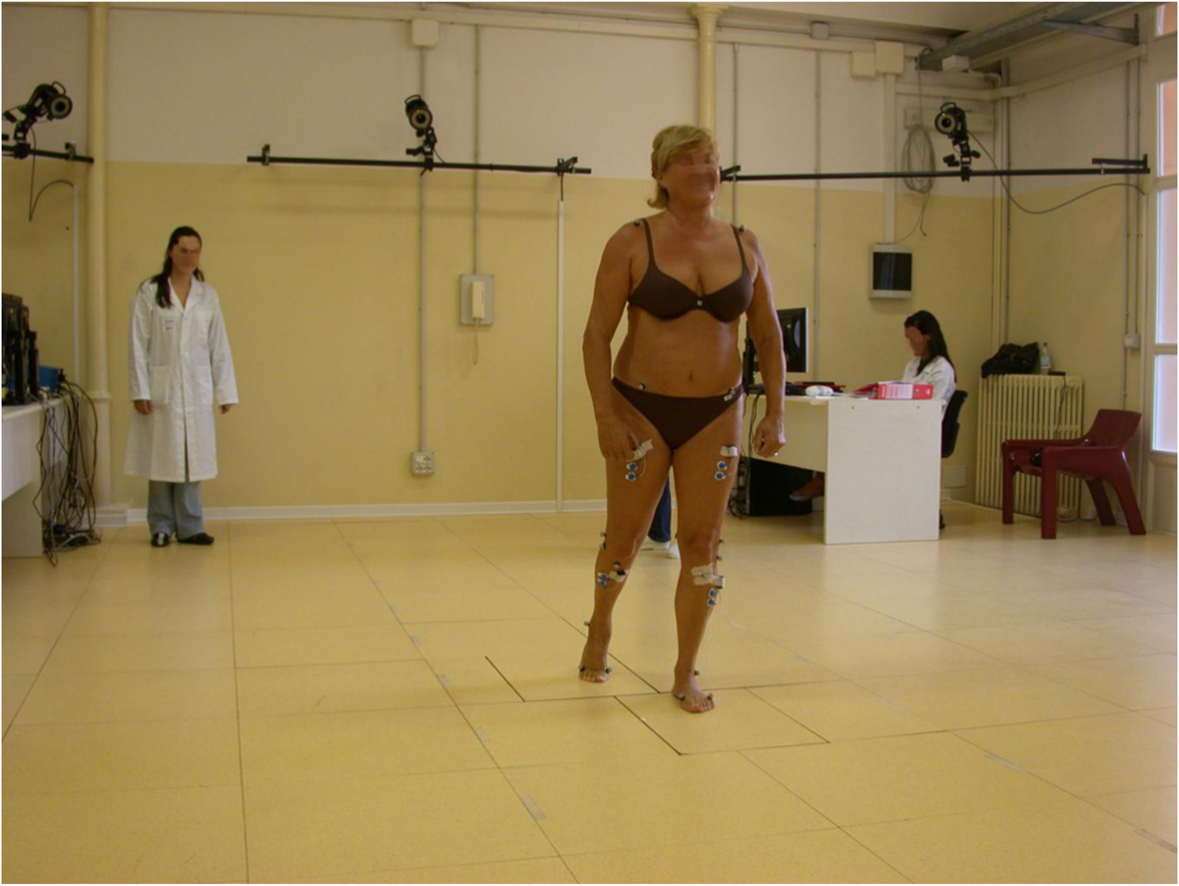
**Standard gait analysis.** A picture taken in the gait analysis laboratory of the authors during data collection for level walking; a patient implanted with TAR is shown. The marker-set is typical of a pelvis plus lower limb motion analysis according to Leardini et al. [38], with three markers only on the foot, considered as a single rigid body, as well as the shank, thigh and pelvis.

**Figure 8:**
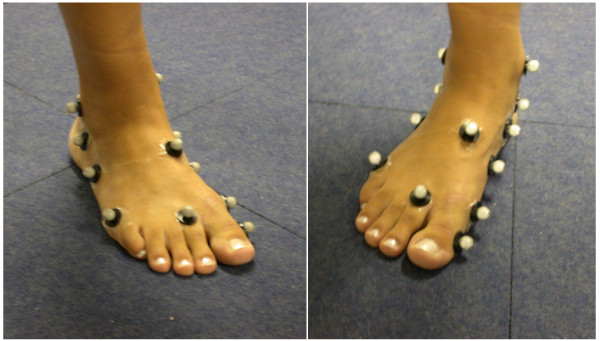
**Marker-set for multisegment foot tracking.** Marker-set for the multi-segment model of foot tracking by Leardini et al. [39]. It includes those three foot markers as in Figure 7.

In standard gait analysis (Figure 7), the foot is considered as a single rigid 3D segment or even a 2D vector in the ‘conventional’ protocol [37] mainly utilised in clinical gait analysis laboratories. The quantitative assessment of normal and abnormal function of the foot and ankle and of the effects of treatment requires an analysis with more sophistication, i.e. a multi-segmental kinematics analysis, able to describe also static deformity and dynamic deformation (Figure 8).

Recent thorough reviews [40,41] classified the known methods of multi-segmental foot modelling, and selected clinical applications of the models. These differ as to terminology, types of the marker-cluster (single skin markers, wands, rigid arrays of markers), 2D- or 3D-based measurements, conventions for joint rotation or planar angle calculation, definition of the anatomical reference frames and of the neutral reference, i.e. the so called offset.

Studies describing these models have shown wide inconsistencies also as to the populations of the healthy subjects analysed, in term of height, mass and age. The most apparent difference however, is for the number of foot segments examined; initially only the rearfoot was analysed, and subsequently mid-foot and fore-foot segments were included in the models, probably because of the availability of more reliable instrumentation.

The most recent studies propose nine- or even ten-segment approaches, although validation in terms of repeatability [42,43] and marker-to-bone association is still limited [44]. Several issues still limit full acceptance and application of these techniques, including visibility, encumbrance, and falling of the markers [10], standardisation of the reports (conventions and terminology), applicability in the presence of severe foot and leg deformities [45], orthosis and shoes [46], and particularly analysis of and possibly compensation for skin motion artefact.

Kinematics in 3D has been assessed also by means of inertial or electromagnetic tracking techniques, although limited to the rearfoot only [47,48]. These sensors require cables but are more practical and definitely cheaper than the stereophotogrammetric systems. On the other hand, the latter can track many different anatomical landmarks on the whole body, whereas the electromagnetic sensors are stuck on the skin over a limited number of anatomical areas of interest.

Other special techniques based on X-rays and on more modern MRI or videofluoroscopy are not applied routinely because of the invasive data acquisition, the restricted field of measurement, and the intense data reduction. However, interesting preliminary studies are developing these methods into accessible clinical applications [49-51], where single foot bone motions can be tracked during activities of daily living. In-vivo skeletal tracking [44] can uniquely provide skeletal motion exactly in standard conditions of daily living activities, but because of the risky invasive procedures it has been limited to physiological motion in a few volunteers. It is definitely inappropriate in routine clinical assessments.

In-vivo foot bone motion has been also mimicked by corresponding in-vitro tracking performed within gait simulators [52-54]. These are highly complicated and expensive systems, but able to replicate in a realistic way overall kinematics, loading conditions and also muscle activation, to be applied to anatomical specimens of the leg. This technique allows access to internal structures and relevant measurements, which is impossible in-vivo, and a few clinical applications are now encouraging their use [55,56]. However the extent to which this replication is reliable has been questioned, repeatability of the measurements is critical, and simulation of the pathological conditions very crude so far.

### Foot and ankle motion in various conditions

Normal foot and ankle motion during locomotion has been reported in many gait analysis reports. It has been shown that, in a population of 20 young normal subjects, about 30 degree rotation in the sagittal plane is coupled to about 14 and 22 degree rotation in the frontal and transverse planes respectively during level walking [57], i.e. a considerable triplanar motion occurs. The critical effect of abnormal foot motion on overall lower limb function has been demonstrated [58,59].

#### In the arthritic ankle

Specific clinical applications of multi-segmental foot models have, in particular, included pathologic gait characterization in rheumatoid arthritis (RA), posterior tibial tendon dysfunction, and hallux rigidus [40]. Woodburn et al. [47] showed that, in these patients, painful valgus deformity of the rearfoot is associated with excessive eversion at the ankle complex and internal rotation of the shank, when walking barefoot and also in shoes. The effect of RA at the forefoot was described by Khazzam et al. [60] by a multi-segmental foot model, supported by anterior-posterior, lateral, and modified coronal radiographs to relate marker position to underlying bony anatomy. As compared to a control population, the RA group showed prolonged stance time, shortened stride length, increased cadence, and a slower walking speed; at the rearfoot, they found delayed and decreased plantarflexion, increased external rotation and increased inversion, in contrast with the previous observations. Turner and Woodburn [61] described RA patients with severe forefoot, rearfoot or combined deformities, and reported decreased plantarflexion in terminal stance and increased eversion at the rearfoot. In particular, they identified different characteristic gait patterns among those groups of patients.

A few papers have compared gait before and after ankle arthroplasty using standard gait analysis, i.e. where the foot is limited to a single rigid segment. Little information is available about gait in ankle osteoarthritis (OA). Two recent studies have described ankle kinematics and kinetics before and after total ankle replacements (TAR) [57,62], and have implied therefore quantitative assessment of gait in arthritic ankles. In the former, with respect to 15 age-/gender-matched control subjects, 15 unilateral post-traumatic ankle OA patients showed a decrease of the second active maximal vertical and the maximal medial ground reaction force, and, at the ankle joint, a decrease of the tri-planar movement, a reduction of the sagittal and transverse moments, a reduction of the power. In the latter paper, 9 patients treated for post-traumatic and 1 for psoriatic arthritis were assessed pre-operatively and at 6 and 12 months follow-up. With respect to the control group as reported at the beginning of 2.2, range of rotation of the foot with respect to the shank in the sagittal, frontal and transverse planes were respectively 11 (30 in normal ankles), 10 (14) and 12 (22) degrees. In both studies, the extent to which the low performance in ankle OA is affected by pain and difficulty in progression is not known, but is demonstrated by low clinical scores and considerable deficiency in most of the spatiotemporal parameters.

#### After arthrodesis

Gait analysis after ankle arthrodesis has been reported only by using a single foot segment model, thus describing, according to the specific marker set, the overall foot-to-shank motion, which includes the confounding effect of foot deformation and the undesirable skin motion artefact [63,64]. Very different motion patterns are expected when either the tibio-talocalcanear arthrodesis (or triple, with intra-medullary nail for a combined ankle and subtalar arthritis) or the isolated tibio-talar fusion (with surgical screws and plates) are performed.

Only two studies were able to distinguish between rear- and fore-foot motion [65,66], although the former paper reported from ten patients only, with no information as to surgical technique and with a large spectrum of follow-up (0.5 – 4 years). In general, the reduced motion at the ankle complex was found to be compensated for by increased motion at the knee and at the more distal foot joints. Significant increase of motion was found radiographically at the subtalar joints in one study [67], stiffness and loss of motion in another [66]. The compensatory hypermobility at the subtalar and midfoot joints is deemed responsible for increased stress at these joints [63,66,67].

#### After total ankle replacement

Since the early 1970s, TAR has been considered a possible alternative for the treatment of severe erosions of the articular surfaces of the ankle, mainly because arthrodesis can result in high incidence of non-union, secondary degenerative changes at neighbouring joints, high incidence of postoperative infection, and total loss of motion [68]. The improving survivorship of ankle replacements and the potential benefits of restoring movement, improving gait and protecting adjacent joints are recent persuasive arguments in favour of arthroplasty [69].

The effect of arthrodesis and arthroplasty, with three different current designs, on the arthritic ankle was analysed preliminary in-vitro [70-72], showing that total replacements changed the natural motion at the ankle joint complex less than arthrodesis, which reduced considerably the range in all three planes as expected. A two-part prosthesis restricted talar motion within the ankle mortise much more than the two three-part designs, likely resulting in an increase of stress forces within and around the prosthesis, potentially leading to polyethylene wear and loosening at the bone-implant interfaces.

In-vivo, gait analysis was performed in a few recent studies. Piriou et al. [69] analysed 12 patients before and after ankle arthroplasty, and compared these to 12 patients after successful ankle arthrodesis and to a healthy control group of 12 subjects. Although neither arthroplasty nor arthrodesis restored normal walking speed or lower limb movements, the former group after arthroplasty had greater motion at the ankle, a symmetrical timing of gait and restored ground reaction force patterns, whereas ankle arthrodesis resulted only in a faster gait with a longer step length compared to arthroplasty.

The two cited studies on kinematics and kinetics analyses in arthritic ankles [57,62], reported on these patients also after TAR. The former described a worsening of gait at three months follow-up, but spatiotemporal variables not different from the normal subjects at 12 months follow-up; however, in six of the eleven kinematic and kinetic variables analysed there was only a partial restoration. In the latter, gait analysis, together with the AOFAS clinical scoring system, was performed at 6 and 12 months from surgery. The function sub-score and the spatio-temporal parameters improved considerably already at 6 months. More normal patterns and ranges of rotations and moments were observed in all the three anatomical planes of the replaced ankle joint at 6 months, and maintained at 12 months. Electromyography revealed also a good recovery of physiological muscle activity. These studies demonstrated that ankle prosthesis can produce an early functional recovery.

Compared to the pre-surgery condition, increased motion at the hip and knee joints, and in ankle flexion moment and power were also observed, at a mean follow-up of four years [73]. Compared to the contralateral non-operated ankle at one year follow-up, several differences were still noted, but nearly physiological motion and loading were observed in the replaced ankle though limited to the stair climbing task [74]. However deterioration of the spatial-temporal parameters and abnormal muscular activation have also been noted at longer follow-up [75].

In explicit comparisons between the two ankle treatments, significantly larger improvements in foot mobility were found after arthroplasty, as expected, with in addition several significant impairments remaining after arthrodesis [48,69,76].

In summary, in-vivo gait analysis showed that although neither arthroplasty nor arthrodesis restored fully normal walking speed or lower limb joint movements, the former allows larger motion at the ankle complex, a symmetrical gait and normal ground reaction force patterns [48,69,73,76], though patients with arthrodesis had faster gait and longer step length [69].

### Mechanics of the stance phase of walking

**Figure 9:**
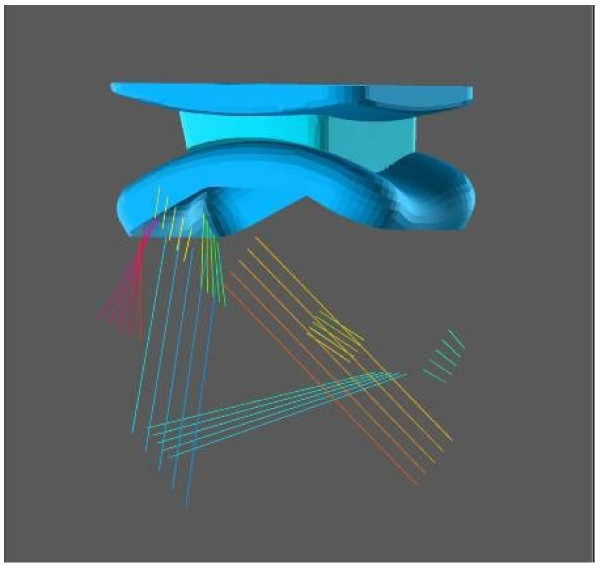
**Diagram at the 3D mechanical model of the replaced ankle.** Three-dimensional mechanical model of a replaced ankle in joint neutral position. Tibial (above), meniscal (in between) and talar (below) components are exactly aligned and fully congruent. Arrangement of the five-fibre ligament model is also shown.

Despite these gait analysis studies, little is known about the inner mechanics at the replaced ankle during daily living activities. A single mechanical model, based on finite element analysis, is available (Figure 9; [77]), which incorporated a previously validated mechanical model of the ankle ligament apparatus and an original three-part TAR. The tibial and talar metal components were modelled as rigid bodies, whereas the intermediate mobile polyethylene meniscal bearing was an elastic–plastic continuum. Overall kinematics, contact pressures and ligament forces were analysed during passive, i.e. virtually unloaded, and active, i.e. stance phase of gait, conditions. Simulation of passive motion predicted similar kinematics as reported previously in an analytical four-bar linkage model for the ankle [78,79]. The predicted patterns of joint rotations were found to be in good agreement with corresponding in-vivo measurements on normal ankles. The meniscal bearing was confirmed to move backward and forward while maintaining full congruity with both the metal components; this contributed to maintain the majority of contact pressures below 10 MPa. In all ligaments, the reaction force calculated from the simulation was well within the known load at failure.

## Current concepts in ankle prosthesis design

Reported unsatisfactory clinical results of TAR [80-85] are accounted for by limited understanding of the mechanism controlling mobility and stability at the ankle and subtalar joints. Relevant 3D models certainly would explain this more realistically, for the benefit of TAR design, but initial models in the sagittal plane only have revealed already fundamental relevant features [78,86] in successful prostheses [87]. The most relevant current issues and the most original current designs in TAR are here addressed.

### Issues in TAR design

TAR designers have been struggling not only with traditional issues in total joint replacement such as materials, fixation elements and techniques, operative techniques, risks of wear and loosening, etc. but also about more recent concepts like joint rotation axes, contact areas, ligaments tensioning, etc. [88,89]. Among these, the following appear to be the most critical.

#### Mobility vs conformity, the dilemma

**Figure 10:**
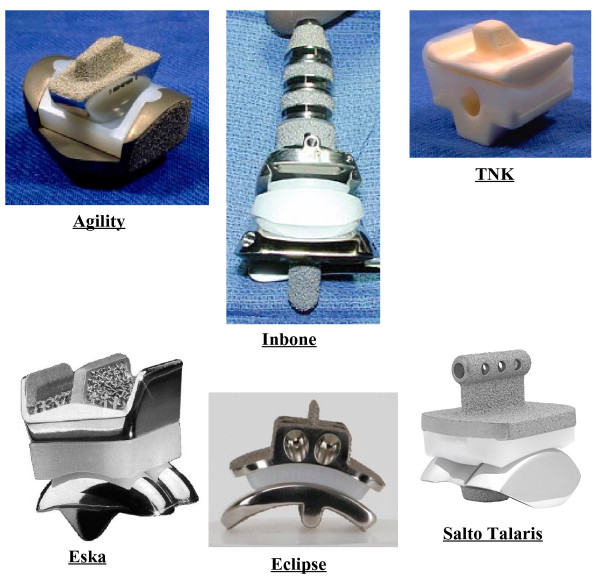
**The 2-part ankle prostheses.** Picture collection of the main current 2-part TAR prostheses.

Total joint replacement must address an original dilemma [3]. When the main target of the designers is the restoration of normal mobility, in terms of patterns and ranges of 3D motion, unconforming, semi-constrained designs (Figure 10) are sought because these allow for the necessary freedom of joint motion; however, this requires incongruent contact with attendant inadequate load-bearing capacity, high contact stresses and eventually high wear rates. On the other hand, when the main target is congruency of the artificial surfaces, full conforming designs are sought, which produce large contact areas which minimises the risk of polyethylene wear, but tend to constrain motion and overload the fixation system. The current generation of three-part TAR designs are the only apparent solution to this dilemma, because the articulating surfaces have conforming shapes, but how the relative motion is guided by the remaining passive and active structures is unknown [78]. Solutions must be sought to guarantee full congruity at the artificial surfaces through the arc of physiological passive flexion.

#### Three- vs two-part prostheses

**Figure 11:**
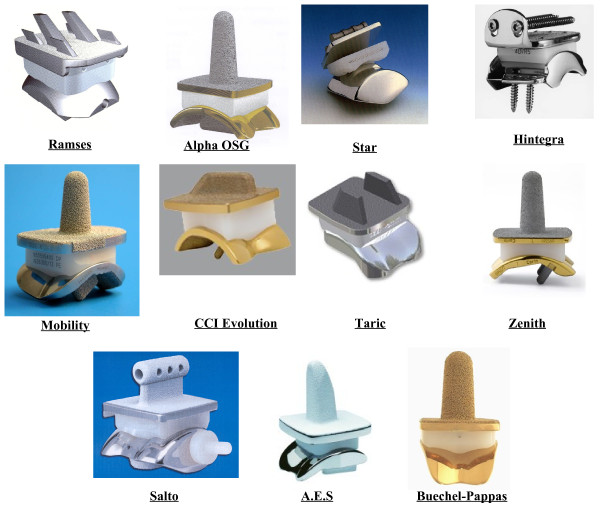
**The 3-part ankle prostheses.** Picture collection of the main current 3-part TAR prostheses.

The implants least susceptible to wear can be completely congruent (or nearly so) two-part devices or three-part designs [81,90] with a meniscal bearing in between the two metal bone-anchored components (Figure 11). The two-part devices require a thick layer of polyethylene typically attached to the tibial component.

**Figure 12:**
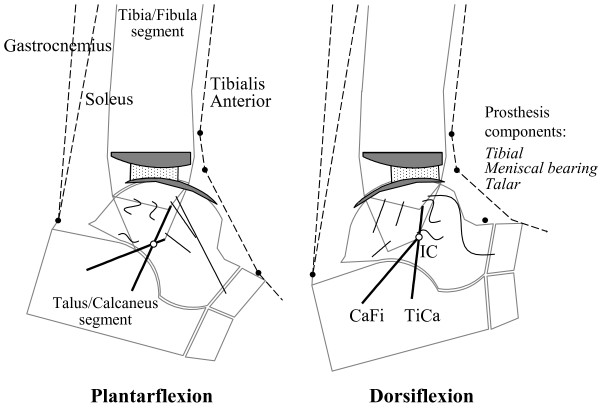
**Diagram for sagittal mobility with the BOX ankle.** Diagram for sagittal mobility of an ankle replaced with the BOX prosthesis. The geometrical arrangement of the passive structures are shown at the extremes of the flexion arc: in maximum plantarflexion (left), and maximum dorsiflexion (right). The kinematics is guided by the isometric rotation of the CaFi and TiCa ligaments (solid bold). The articular surfaces (the arcs in nearly contact), the other ankle ligaments (buckled segments), and the instantaneous centre of rotation IC (empty circle) are also depicted. With respect to Figures 1 and 2, the course of the three main muscle-tendon units and the pulleys (full circles) representing the extensor retinaculum bands for force redirection are also depicted. The bi-concave meniscal bearing (dots area, in between) is required to slide forwards on both components during dorsiflexion and backwards during plantarflexion so that the bones roll as well as slide upon each other. During this motion backward and forward, full congruity is maintained at the two articulating surfaces. The rolling element of the relative motion is manifested by the sliding of the bearing on the tibial component. The axis of dorsi-plantarflexion passes through IC and moves forwards and proximally during dorsiflexion, backwards and distally during plantarflexion.

The three-part designs employs fully-congruent meniscal bearings free to slide on both the articular surfaces of the component fixed to the bones. A meniscal bearing prosthesis can allow translational movement and yet maintain congruence of all the articular surfaces throughout the range of movements. One of the two bone-anchored components must have constant radius to allow fully-congruent contact with the meniscal bearing in all joint positions. To also allow translational movement, the other component should have a flattened surface, although slightly concave or convex fully-congruent surfaces can also be used. Flat tibial components can experience only compressive force assuming no friction, and therefore these would transmit only compressive stress to the bone-implant interface, and would not need a robust intramedullary stem for fixation to the bone. A polyethylene meniscal bearing component is inserted in between, with the articulating surfaces fully-congruent to those of the metal bony-anchored components. It is free to translate in any direction to accomplish the relative movements of the two components as guided and constrained by the passive and active structures at the joint (Figure 12). Dislocation of the meniscal bearing is resisted by the interpenetration of the convex bony-anchored component into the concavity of the bearing, in which it is held by the tension in the joint’s ligaments. A spherical interface between the convex bony-anchored component and the meniscal bearing has also the advantage of maintaining congruence also in transverse and frontal plane rotations. Unlike a cylindrical interface, the spherical interface can also accommodate for slight inaccuracies of implantation. Intact retained ligaments can be restored to their original normal tensioning pattern by the choice of an appropriate thickness of the bearing component. As in the natural joint, as shown above, where articular surfaces alone do not guide the reciprocal movements of the bones but merely allow them, in meniscal bearing replacement the unconstrained components perform in the same way as guided by the ligamentous mechanism.

All these considerations emphasise the importance of the intact status of the ligamentous structures in any ankle meniscal bearing replacement. The implantation of freely mobile bearings into joints which lack an intact and functioning ligamentous apparatus is theoretically mistaken, and has proven to be unsatisfactory in practice also for knee replacements [91]. It is irrational to build into a prosthesis the freedom to translate in the absence of the mechanism which controls that freedom.

Currently most of the TAR designs in clinical use have fully conforming mobile bearings [89], and only apparently these represent correct compromises between mobility and conformity. These are claimed to be anatomical, but all feature a flat shape of the tibial component, very unnatural, in addition to the natural anatomical talus. These must rely fully on ligaments for final joint stability, but unfortunately the functioning of the ligaments was not considered explicitly in the design.

#### Implantation, fixation and materials

In addition to replication of original joint function, i.e. mobility and stability, it is also necessary to achieve implantability and durability in TAR. The reliability and repeatability of the operative technique is considered by the surgeons as a fundamental characteristic for a TAR design. Relevant instrumentation must be robust and accurate enough for guaranteeing the correct position of the components with the minimum bone stock removal. Durability is also dependent on good fixation of the components, which would involve an appropriate load transfer to the bone and a minimum risk of loosening. The current designs show a large variety of fixation elements. Pegs, long or short stems and cylindrical or rectangular bars have been used [92]. More recent designs use bone screws [93,94].

As far as the materials are discussed, moving from the original tibial components made in polyethylene, most of the recent two-part designs include a metal-backed tibial component. The design of the elements used to limit the floating of the bearing core is then an additional critical issue. Entrapment of the meniscal bearing in some prostheses is enforced by sharp limiting interfaces, to prevent dislocation and separation. Ribs and grooves, lugs and cutouts, and even systems of interlockable flanged grooves have been devised for this purpose [83,84,94]. These latter prostheses may be at high risk of polyethylene wear through contact at these interfaces.

### Current and future developments

From the numerous reviews of the current TAR designs [83,84,89,94], it emerges that only a few different conceptual approaches have been followed. Basically, in the two-part devices, the replication of the original anatomy was sought. On the other hand, in the three-part designs, the introduction of a non-anatomical meniscal bearing, flat above and nearly anatomical below, was assumed to achieve the necessary conformity.

The TAR design formulated by the present authors was the first in which the shapes of the articular surfaces in the sagittal plane were chosen to have a natural interaction with the retained ankle ligaments [78,79,86]. The design process followed investigations [17,21,22] which included measurements on cadaver specimens in virtually unloaded conditions and mathematical models. These have shown how the mutual action of the passive structures of the ankle control and limit joint motion, i.e. articular surfaces and ligaments interact together in a complementary and mutually compatible manner. A feature of the surface/ligament interaction which the new design attempts to reproduce is to allow fibres within the calcaneofibular (CaFi) and tibiocalcaneal (TiCa) ligaments to remain isometric over the range of passive motion while all other ligament fibres are tight only at the limits of plantar- or dorsi- flexion.

Previous designs of TAR focused exclusively on the geometry of the prosthetic components in relation to the morphological features of the intact articular surface of the talus [92,95,96]. Our mathematical analysis (Figure 10) showed that the fixed articular surfaces should both have anatomical shapes or should both be non-anatomical [78]. Current three-part prostheses [93,97-101] use a more or less natural-like convex surface for the talar component and a non-anatomical flat surface for the tibial component. This combination of anatomical and non-anatomical surfaces cannot be compatible with the retained ligaments [78]. Early clinical results suggest that a ligament-compatible TAR design can achieve good clinical results [87], a low wear rate [102] and a good recovery of function [57]. Direct comparisons with other TAR designs and longer term outcome studies are required to corroborate these short term observations.

Recently, there has been renewed interest in ankle joint replacement likely because longer term outcome studies have become available, and because the FDA has approved a few more designs in the United States [83,103], for the options for TAR surgeons being greatly expanded. Most recent efforts in TAR development seem to be dedicated to two-part devices, apparently under the assumption that the failure of the original such designs was due only to the poor quality of the fixation elements and of the polyethylene inserts. Despite the general tendency in orthopaedic surgery to simpler and quicker surgical procedures, most recent designs seem to require long techniques and cumbersome apparatus [83]. In addition to optimal component design, there continues to be much debate within the surgeons interested in TAR as to indications, patient selection, and operative technique.

## Conclusions

The mobility and stability of the ankle joint have been investigated extensively, but many critically important issues still need to be elucidated. However, there seems to be a general agreement on several important observations. A more isometric pattern of rotation for fibres within the calcaneofibular and the tibiocalcaneal ligaments with respect to all the others has been shown. Many recent studies have found changing positions of the instantaneous axis of rotation, suggesting that the hinge joint concept is an oversimplification for the ankle joint. A few recent works have also claimed anterior shift of the contact area at the tibial mortise during dorsiflexion, which would imply combined rolling and sliding motion at this joint. Many findings from the literature support the view of a close interaction between the geometry of the ligaments and the shapes of the articular surfaces in guiding and stabilising motion at the ankle joint. Any design of joint replacement or ligament and articular surface reconstructions must take into consideration these important findings.

## Abbreviations

2D: Bi_dimensional; 3D: Three-dimensional; CaFi: Calcaneofibular ligament; CN: Common normal; IC: Instantaneous centre of rotation; TAR: Total ankle replacement; TiCa: Tibiocalcaneal ligament.

## Competing interests

The Istituto Ortopedico Rizzoli and John O’Connor received royalties for the intellectual properties of the BOX Ankle device, that described in Giannini et al. [84].

## Authors’ contributions

AL carried out most of literature review work, and drafted the manuscript. JJOC contributed to the original organisation of the manuscript and edited its final versions. SG participated in the discussions about the anatomical, surgical and clinical issues, and contributed to the right interpretation of the clinical studies from the literature. All authors read and approved the final version of the manuscript.
